# The white matter of the human cerebrum: Part I The occipital lobe by Heinrich Sachs

**DOI:** 10.1016/j.cortex.2014.10.023

**Published:** 2015-01

**Authors:** Stephanie J. Forkel, Sajedha Mahmood, Francesco Vergani, Marco Catani

**Affiliations:** a University College London, Department of Psychology and Language Sciences, Research Division of Clinical, Educational and Health Psychology, London, UK; b Natbrainlab, Department of Neuroimaging, Institute of Psychiatry Psychology and Neuroscience, King's College London, London, UK; c Department of Neurosurgery, Royal Victoria Infirmary, Newcastle upon Tyne, UK; d Institute of Neuroscience, Newcastle University, Newcastle upon Tyne, UK; e Natbrainlab, Department of Forensics and Neurodevelopmental Sciences, Institute of Psychiatry Psychology and Neuroscience, King's College London, London, UK

**Keywords:** White matter, Heinrich Sachs, Wernicke, Post mortem dissection, Occipital lobe

## Abstract

This is the first complete translation of Heinrich Sachs' outstanding white matter atlas dedicated to the occipital lobe. This work is accompanied by a prologue by Prof Carl Wernicke who for many years was Sachs' mentor in Breslau and enthusiastically supported his work.

## 1 Introduction

Born in 1863, Heinrich Sachs was a German neurologist and neuroanatomist who obtained his specialisation in neurology and psychiatry with Carl Wernicke in Breslau (Forkel, 2014). Sachs published on amyotrophic lateral sclerosis (1885), aphasia (1893; 1905), and traumatic neurosis (1909), but arguably his most distinctive contribution was in the field of white matter neuroanatomy. Whilst still a doctor in training he spent most of his time looking at series of cross-sections obtained from human brains. This painstaking effort resulted in the publication of the first atlas of the occipital lobe connections in the human brain (Sachs, 1892). Sachs's atlas contains detailed descriptions of the methodological approaches he employed, which makes the text not always an easy reading; but the figures are beautifully informative and include many previously undescribed tracts. For example, Sachs defined three different layers in the deep white matter of the occipital lobes, which he correctly identified as fibres of the splenium (forceps corporis callosi, see page 15), optic radiations (stratum sagittalis internum, see page 18) and the association pathways (stratum sagittalis externum, see page 20). These were later indicated with the name *stratum sagittalis of Sachs* in recognition of his work. He also introduced a new nomenclature for the vast number of U-shaped fibres running near the cortical surface of the occipital cortex. The knowledge of these tracts had direct clinical relevance as differences between apperceptive and associative visual agnosia could be explained in terms of primary visual cortex damage and damage to associative U-shaped fibres, respectively (Lissauer, 1890). In contemporary neuroscience we have understood that within the occipital lobe these U-shaped fibres mediate crosstalk between the ventral visual stream dedicated to objects-perception (the ‘what’ pathway) and the dorsal visual stream dedicated to place location and motion perception (the ‘where’ pathway).

Sachs' mentor Wernicke was an enthusiastic advocate of his anatomical insights and encouraged his trainee to further pursue this research. The atlas was in fact intended to be a multi-volume project in which subsequent books would have been dedicated to the function and clinical correlates of each tract. This was an ambitious project in the footsteps of the great clinical anatomists of the time. Unfortunately, Sachs did not complete what he had set out to accomplish and never returned to his master plan in the four decades he continued working as physician at the neurology and psychiatry clinic in Breslau.

Despite its importance, Sachs's atlas went unnoticed for decades. This is in part due to the availability of more detailed information on connectional anatomy derived from axonal tracing studies performed in animals. Also the lack of an integral translation from German to English did not facilitate its dissemination.

We believe that with the advent of novel MRI-based methods to study connections in the human brain, the work of Sachs could be of great relevance to contemporary neuroscience. This is particularly true for those tracts that may underlie uniquely human abilities. The vertical fasciculus of Wernicke, for example, connects relevant areas for reading. Sachs describes this tract in detail and credits his description to Wernicke (see page 26). Despite this tract being one of the largest intraoccipital connections, its function has remained unknown. More recent studies in patients with lesions to this white matter tract or its cortical projections suggest that it may have a role in reading (Yeatman, Rauschecker, & Wandell, 2013). Other tracts described by Sachs are still waiting to be ascribed a specific functional correlate.

Sachs's occipital tracts have been recently replicated using post mortem Klinger dissection (Vergani, Mahmood, Morris, Mitchell, & Forkel, 2014). Detailed tractography studies are needed to characterise the in vivo anatomy of these tracts in terms of interindividual variability as previously shown for tracts of other lobes (Catani et al., 2007; 2012; Forkel et al., 2014; Lopez-Barroso et al., 2013).

## 2 Translation

In Memoriam to Dr. Heinrich Lissauer, Assistant at the psychiatric clinic in Breslau. Publications from the psychiatric clinic in Breslau.

“The white matter of the human cerebrum. Part 1. The Occipital Lobe” by Dr. med. Heinrich Sachs, neurologist in Breslau with a prologue by the medical officer of health Prof. Dr. C. Wernicke, including 3 figures and 8 plates.

## 3 Prologue by Wernicke

The present work is the first contribution to a series of publications dedicated to the investigation of the brain and its functions in health and pathology. This field of research is still heavily under investigated and nearly every contribution to it is a step forward similar to an expedition into unknown territory comparable to the “deepest Africa”. The integration of clinical observations and anatomical aspects has constantly proven to be a reliable method to move forward. The advances in anatomy, which are naturally slow, will be followed promptly by our clinical experience.

The anatomy of the white matter of the cerebrum always intrigued me as the link between all delicate clinical methods; hence, I appreciate with great satisfaction that our colleague Sachs made such an encouraging start with the present work, which is of the highest standard in terms of its content and structure. May future publications be equally well received by colleagues. Breslau, January 1892.

## 4 Sachs' atlas

This work can be considered as the first part of a more extensive work on the white matter fibre trajectory in the healthy adult human brain. The dissections presented here were obtained in the psychiatric clinic in Breslau. I shall take the liberty to express my gratitude towards Professor Wernicke for kindly granting me permission to undertake this work and for his suggestions. Further, I thank the assistant, Dr. Lissauer, for his friendly and active support.

The aim of the work is to provide a macroscopic overview of the fibre connections of the occipital cortex as well as adjacent parts of the parietal and temporal lobes. Details and subtleties can be added to this work in the future.

Information on the white matter anatomy of the cerebral hemisphere is relatively scarce. In order to gain an overview of this field one has to go back to the beginning of the century, namely to Burdach's great work (1819; 1826; 1822), as fibre trajectories are only hinted at in more recent textbooks. The work by Meynert (1884) is difficult to understand and is not entirely evidence-based. Furthermore, the available case reports are based on pathological specimens. Foundation work demonstrating the white matter anatomy in the healthy adult brain is entirely missing. However, in order to assign each case report its apt place in the system, the healthy human brain should be the reference for all other studies of pathological, foetal, and animal brains.

Identifying the directionality and trajectory of fibres within the white matter using only a single method is insufficient as each method has its inherent limitations. In order to overcome these limitations the results of at least two methods ought to be compared. For the current study three complementary methods were therefore employed:

1. The oldest method is “Zerfaserung” [post mortem blunt dissection], which was used exclusively by Burdach and honoured in particular by Meynert and his students. For the current work, I used brains that were treated with alcohol, yet were not too hardened. The method introduced by Stilling (1882) which uses “Holzessig” [wood vinegar] returns brilliant results for the brain stem but was, however, not suitable for the white matter of the hemispheres. The difference is that for this work it is not important to segment small parts of the brain into its fibre pathways but to relate the overall direction of fibres and connections between white matter bundles within a lobe. In contrast, blunt dissections return perfect results if the majority of fibres are running along the same direction, whilst the ubiquitous crossing fibres are not forming substantial bundles but are present in isolation or small numbers when piercing through the main pathways. In such cases they would fall apart smoothly or one does not notice them at all unless already familiar with them. Additionally, the presence of large fibre crossings can be identified using this method. However, it is not possible to follow with confidence the trajectory of the fibres beyond their point of convergence. Further, if fibres that thus far run in parallel start adhering to each other, as it is the case for callosal fibres towards the midline, this method will also fail. In both these cases tearing the tissue can create arbitrary artefacts. Coarse crossovers are not found in the occipital lobe and matting [occurs] only in the corpus callosum.

The most important drawback of the method is that it only gives us two-dimensional views. The direction and the width of a layer can only be identified with certainty if the layer is entirely destroyed. Therefore, blunt dissections are only for demonstration – in this case they are obviously invaluable to appreciate special organisation and relationships – but they are never sufficient as evidence in their own right.

2. The second method is the inspection of freshly prepared sections of specimens hardened in Müller solution and observed under direct light. These sections show the fibres or layers cut horizontally as pure white with only a hint of green. Areas where fibres are cut obliquely appear black-green and are darker in their shade than the dark green colour of the grey matter. Between these two extremes all shades of colours can be found depending on the cutting angle in relation to the direction of the fibres and whether the majority of the exposed fibres were cut straight or oblique in regions of multiple fibre orientations. Additionally, differences in fibres, such as their width or the chemical nature of the myelin sheath, influence the tone of colour, so that the various layers can be clearly differentiated. If the brain is cut along different directions, the same layer can appear light or dark depending on the direction of the cutting.
3. The most important method, that allows magnification and inspection of individual fibres is the method introduced by Stilling and perfected by Meynert, where a series of sequential translucent and stained sections is prepared. To my knowledge only Pal's Haematoxylin is suitable as stain. In comparison, when sections of 1/20 mm thickness are prepared using Weigert's stain the white matter appears evenly blue, thus making it impossible to differentiate the various layers. The preparation of sections as thin as 1/10 mm for the whole hemisphere was only possible with great effort [as it requires], time and skill. Extremely rare is the successful transfer onto a glass plate after the specimen was exposed to the strains of staining and brightening. In contrast, 1/10 mm sections were suitable for staining according to Pal. Pal's stain has the advantage of staining some fibres very dark and deep blue, whilst other fibres are only lightly stained in brown-glow. Hence, it is possible to differentiate them. In the brainstem five different shades of colour can be distinguished. These shades depend partly on the variable thickness of the fibres and partly on the chemical properties of the myelin sheath. Another advantage of this method is, that sections stained with this method can be photographed. The photographs enclosed to this work [8 plates at the end of this publication] are an example of such sections and were provided by Dr. Lissauer.

The Picro-carmine stain allows identifying various white matter layers with the naked eye and the nuclei can be seen under the microscope. Structures that are usually coloured dark and blue by Pal's stain are stained yellow by picro-carmine. What appears light and brown using Pal is reddish with picro-carmine. The drawback is that in brain tissue, unlike peripheral nerves or cord, the axonal cones are not distinctly stained in red; therefore the individual fibres cannot be differentiated.

Note: When using Pal's stain for large specimens, such as a section of the whole hemisphere, a multitude of stratagems are required and negligence of each of them endangers the final result. I shall therefore carefully describe the method below.

The brain is removed from the skull as soon as possible after death, ideally in the winter and then preserved in Müller solution as a whole or only cut in halves (to avoid losing its shape). In the first few days, the solution needs daily changing. The specimen is ready to be cut after three to four months. Slices, cut as thin as the microtome allows, are dried by soaking them in diluted alcohol and pure alcohol, each for a period of 24 h. Slices are then immersed in celloidin solution and stuck to wooden plates. For the sections I used the largest Schanz microtome and an especially designed heavy knife. I did not cut under spiritus. Slices of 1/10 mm thickness can be picked and transported easily if not yet stained. If the brain is rather crumbly, the surface can be covered with collodion or celloidin by dripping on a thin layer of the solution prior to each cut. The slices are placed – without copper – in water for 24 h and subsequently in a 1% haematoxylin solution (Haematoxylin 1, alcoh. Abs 5, of which 5 ccm onto 100 ccm water and 1 ccm saturated lithium carbonium solution) for the same length of time. One can simultaneously stain 10 or 20 slices in a large amount of solution, but the same solution cannot be used twice. The slices are then washed with plenty of water and de-stained; it is best to let them soak in water for a period of 24 h. They can, however, be left in water for longer without any concern; in which case the slices only de-stain faster. The individual slice is then placed onto a glass plate or in a glass dish with fatty margins and is poured onto with a .5–1% manganese-rich acidic potassium solution and gently turned around multiple times. The solution has to be changed repeatedly and is only actively de-staining as long as it shimmers bluish when held against a white paper. As soon as the blue colour is changing towards violet, the solution does not de-stain any longer. On the contrary, it rather stains permanently brown. Consequently, if the section is not strongly dehydrated following the stain and contains rests of free haematoxylin, one swiftly has to discard the first portion of the potassium solution. In cases where the margin of a section is transparent and free of black stains when it is held against sunlight or a bright flame, the section is carefully washed with water and poured onto with an acidic and ideally hot solution (Ac. Ocalic. .5, Nat. sulfuros. .5, Aq. 200). The section is then gently swung in the solution until the margin is perfectly white and stain free. If necessary, the acid solution can be changed. Should stains still persist, one has either the option to be satisfied with the result or otherwise restart the process with potassium solution after washing the slice in water. A repetition is also advisable if the staining was very intense and the layers are thus not distinguishable after the first staining. In such a manner, de-staining can be carried to the extreme. The more de-staining is carried out the brighter the entire slice becomes. This however also applies to the delicate fibres, especially cortical fibres, which can be de-stained to the point where they will fade. If a slice that is too bright and brown it can be stained darker and blue when covered in alkaline solution, an ammonia solution or carbonic lithium. The slice – from now onwards placed on an object slide – is dried in absolute alcohol and the celloidin is removed with ether alcohol.

If the slice was covered with celloidin prior to cutting, it is best to make sure that the side of the slice that was covered with celloidin is placed facedown on the stage. It is then lightened in carboxylox (ac. Carbol. 2. Xyl.6.). One drains the carboxylol a little and presses at least eight layers of blotting paper quickly and strongly on the slice. The uppermost page of blotting paper should not become wet, as parts of the slide will stick to it. The slice is then poured over with warm or Xylol-thinned Canada-balm and covered with a thin glass plate. During microscopy, it is best to look without aperture using an Abbé microscope.

**Fig. 1:**
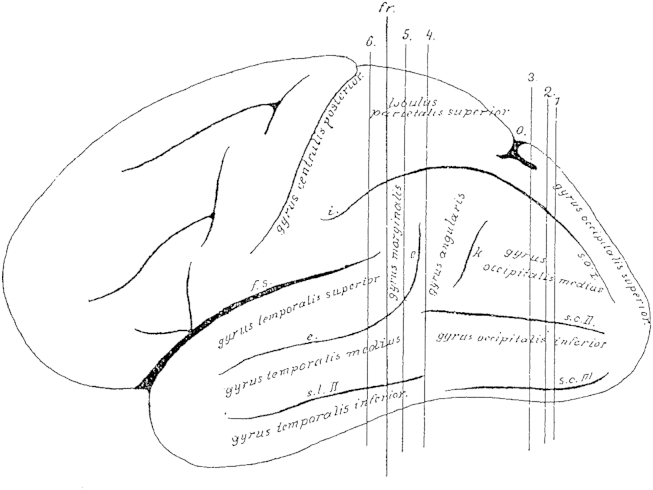


**Fig. 2:**
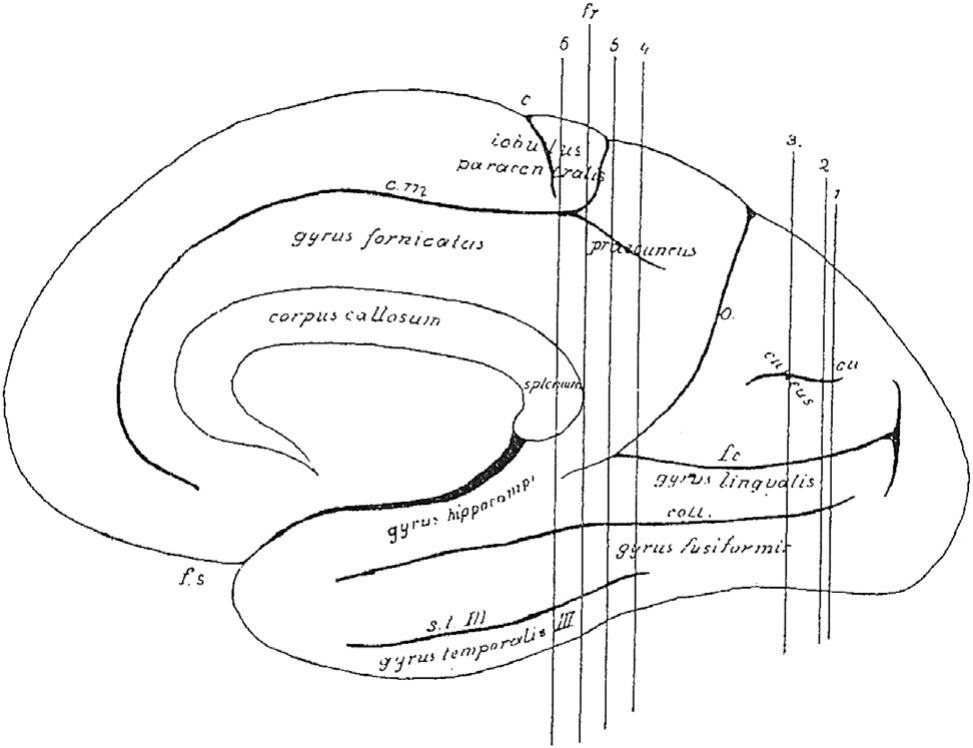


The cortex, whose white matter connections are to be described here, is delimited anteriorly by a frontal plane [fr], which passes tangential to the posterior end of the splenium (Figs. 1 and 2). The natural boundary for the white matter of the occipital lobe, the confluence of the posterior horn in the *cella lateralis* of the lateral ventricle – the opening of the posterior horn – lies just behind this plane.

On the convexity of the medial surface this plane cuts the most anterior part of the precuneus (Fig. 2). On the lateral convexity (Fig. 1) it cuts the gyrus at the end of the Sylvian fissure [supramarginal gyrus], whose most posterior cortical indentation extents into the depths. On the lateral convexity of this three-sided piece of brain, two sulci can be seen running dorso-ventrally [e,k], and three sulci running posterior-anteriorly [s.o. I–III], which all impact on the shape of the underlying white matter due to their depth. The more anterior sulcus [e] of the two vertical sulci is the ascending branch of the parallel sulcus [superior temporal sulcus] (Fig. 1e). This ascending branch lies entirely within the parietal lobe and is considered as part of the angular gyrus. The adjacent posterior vertical sulcus is the anterior occipital sulcus [posterior intermediate parietal sulcus] (k; see Wernicke (1881)). This sulcus considered representing the border between the parietal and the occipital lobes. This sulcus can appear in different shapes. Usually, it continuous ventrally into the continuation of the superior temporal sulcus [e] and thus gives rise to a second ascending branch of the latter. At times, however, it appears as a very short indentation without connection to any other gyri. It is, nonetheless, found in every brain and is readily identifiable, when following the occipito-parietal sulcus (o) on the convexity (Fig. 1) to the inferior transitional gyrus (above k) (Fig. 1) between the parietal and the occipital lobes. The opening of this gyrus is the anterior occipital sulcus.

Within the occipital lobe there are three deep sulci that are almost horizontal to each other before they separate anteriorly (Ecker, 1869). The superior/first occipital sulcus (s. o. I) is an extension of the intraparietal sulcus (i), which usually reaches the occipital pole, though interrupted. The middle/second occipital sulcus (s. o. II) reaches anteriorly towards the horizontal branch of the superior temporal sulcus (e). The inferior/third occipital sulcus (s. o. III) runs towards the second or third temporal sulcus. The inferior occipital sulcus often runs adjacent to the inferior convexity of the hemisphere and sometimes even at the basal surface. The middle occipital sulcus corresponds mostly to the lower occipital sulcus of Wernicke. Whereas both vertical sulci and the first horizontal sulcus are consistent and readily identifiable; the middle and inferior occipital sulci are often interrupted and branch off, and are therefore less clear.

**Fig. 3:**
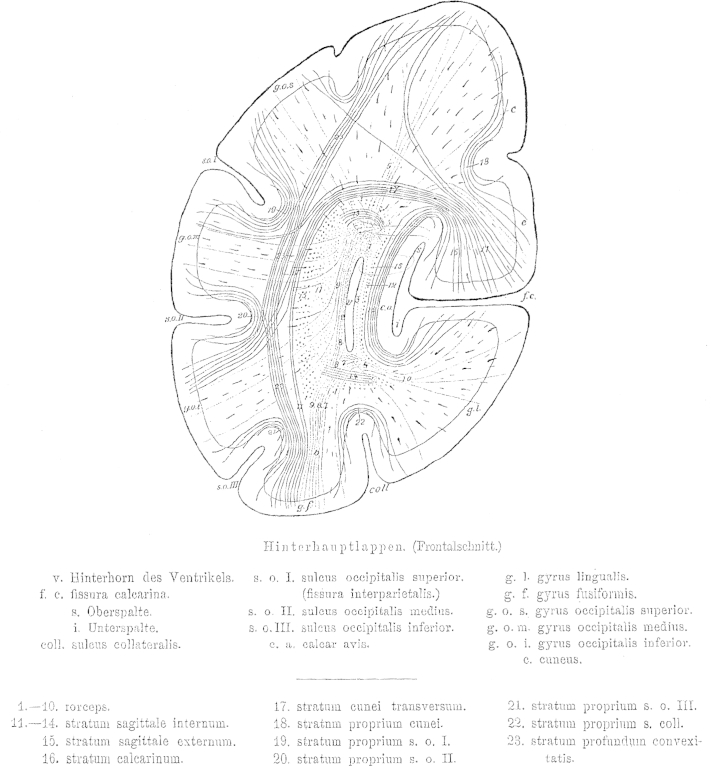


The occipital lobe is delineated on the medial surface of the hemisphere (Fig. 2) by the occipito-parietal sulcus [o] separating the cuneus and precuneus, and by the calcarine fissure (f.c.), which adheres anteriorly with the above-mentioned sulcus [o]. Both sulci are rarely simple incisions. Usually, their stem forms a surface similar to the insula with secondary gyri. Nevertheless, this morphology is variable. The “posterior incision” of the occipito-parietal sulcus may extend many centimetres into the occipital lobe. Adjacent to the calcarine fissure a short gyrus extending rostro-caudally can be seen superimposed on the top and bottom surfaces facing each other. In the depth of the fissure three vertical short gyri extend dorso-ventrally. Two of these can continue to the convexity of the sulcus and merge with the above-mentioned gyri; whereas the third sulcus, that is the middle or the posterior, never extends to the convexity. Such a short gyrus can reach at times the convexity and thus interrupt the fissure. The calcarine fissure, similar to the Sylvian fissure, has a superior (s) and inferior (i) opening (Fig. 3) but no posterior extension. Rather the base of the fissure becomes flattened and continues onto the medial surface of the hemisphere. This transition is to be seen in the forking of the fissure posteriorly. Within the cuneus, a gyrus parallel to the calcarine fissure extends rostro-caudally [cu, Fig. 2].

In the precuneus, the horizontal, posteriorly directed extension of the sulcus calloso-marginalis (cm, Fig. 2) [cingulate sulcus] is important for white matter anatomy.

On the basal surface, the most important sulcus, which shapes the white matter is the collateral sulcus (coll., Fig. 3), which is the fifth sulcus to extend caudo-rostrally between the calcarine fissure and the inferior occipital sulcus and is variable in its extension in both directions. The medial occipito-temporal sulcus reaches very closely the occipital pole. In cases where the calcarine sulcus is a simple incision, the medial occipito-temporal sulcus can present a complex division.

The occipital horn begins to form as a canal with four walls, with thin dorsal and ventral walls and two-to-four-fold wider medial and lateral walls. Posteriorly, it rapidly looses its shape in all directions. Initially the loss is primarily in height more than width, so that it resembles almost a square before it looses its width and thus becomes a thin sulcus with its dorsal and ventral walls turned into edges. During its course it bends posteriorly in two directions. In its posterior part it bends gently along a vertical axis and thus its posterior end comes to lie closer to the medial plane than its aperture. In addition, it bends along a sagittal axis and becomes a slit, thus bringing the dorsal and ventral edges closer to the medial plane. From its posterior end a strip of ependyma, which retains its form, continues into the occipital white matter for a short distance. The double bend of the horn resembles the form of the hemispheric convexity and is due to the deep [occipital] notch close to the calcarine fissure. Apart from this, only the medial occipito-temporal sulcus has an impact on the shape of the occipital horn, by bulging its inferior surface a little in the middle part of the horn. All the other sulci, including the secondary deformations of the calcarine fissure, are of no importance to the shape of the occipital horn. These influence the width of the white matter only, and as is later to be seen, the thickness of the fourth and outermost layer, which lies immediately underneath the cortex and is referred to as the stratum proprium cortices. The deeper layers of the white matter are independent of the depth of these sulci.

The occipital horn lies closer to the basal surface than to the dorsal convexity of the hemisphere (Fig. 3); yet, it is equidistantly located between the medial and lateral surfaces. Nevertheless, due to the depth of the calcarine fissure it is separated from the cortex at the medial surface by a thin layer of white matter. The majority of white matter, on the other hand, develops at the lateral aspect of the occipital horn between the latter and the hemispheric convexity.

The fibres originating from the occipital cortex and coursing within the occipital white matter can be divided into two groups. Amongst these groups, one can again subdivide three groups: i) fibres that extend to subcortical centres and are considered as projection fibres or corona radiata (Stabkranz) (Meynert); ii) other fibres have their terminations in cortical areas and are therefore association fibres. Association fibres either interconnect intralobal cortical areas (short association fibres), or link the occipital cortex with the cortex of a different lobe (long association fibres); iii) the third group crosses the inter-hemispheric midline and might terminate in [contralateral] cortical or subcortical areas (callosal or commissural fibres).

This mass of the occipital lobe fibres is not a tethered bundle, but is rather organised into bundles and layers according to certain rules. These layers can be distinguished based on their direction, grouping and staining. The law of order is the following (Wernicke as cited above, p. 24): Every fibre reaches its destination via the shortest possible route, as far as this is in correspondence with embryological peculiarities of brain development. Thus, the following two conclusions can be reached: First, short fibres are located close to the cortex whilst longer fibres are located close to the ventricle. Second, fibres with roughly the same destination run in parallel or form bundles for a part of their common trajectory.

A second, generally valid biological law not to be ignored in the study of brain structure is the law of variability. There are no two brains that are identical in all their details. Variability is also observed in the arrangement and development of white matter anatomy. The cortex and the white matter are mutually dependent on each other. If a particular area of cortex is under-developed in a brain, then there is also a paucity of fibres originating from this area.

The occipital lobe fibres form four layers, which envelop the occipital horn like an onion skin from all sides except its opening. These layers, counted outwards from the medial to the lateral walls of the ventricles are (Fig. 3):

1. Layer of the corpus callosum: Forceps corporis callosi (1–10), a. pars magna superior (1), b. pars parva inferior (4)
2. Layer of the projection fibres: Stratum sagittale internum (11–14)
3. Layer of the long association fibres: Stratum sagittale externum (15)
4. Layer of the short association fibres: Stratum proprium cortices.
  a. stratum calcarinum (16)
  b. stratum transversum cunei (17)
  c. stratum proprium cunei (18)
  d. stratum verticale convexitatis.
    α. stratum proprium fissurae interparietalis see also sulci occipitalis I. (19)
    β. stratum proprium sulci occipitalis II. (20)
    γ. stratrum proprium sulci occipitalis III. (21)
    δ. stratrum profundum convexitatis (23)
  e. stratum proprium sulci collateralis (22)
  f. stratum proprium praecunei.

This layer is found in the region of the parietal lobe.

Another two bundles are closely located to the occipital lobe without joining its white matter system, namely:

5. Bogenbündel or oberes Längsbündel, fasciculus arcuatus see also longitudinalis superior [superior longitudinal fasciculus]
6. Zwinge, cingulum.

Fibres originating from the occipital pole and surrounding areas bundle up in the middle of the white matter and run anterior-posteriorly. Far before the ventricular horn, these fibres group together into three concentric layers. The inner solid layer gives rise to the forceps. The remaining layers, which on axial sections look like a ring, form the internal and external sagittal layers. The forceps appears clearly as an independent layer, a few millimetres anterior to the other two layers. Thus, in the small stretch that includes the stratum proprium corticis, only three layers are evident. The three layers with a sagittal direction thicken anteriorly as new cortical fibres from all directions join them. At the caudal end of the posterior horn these fibres part like a funnel, so that all three layers equally cover the posterior horn.

## 5 Forceps corporis callosi

If there was no posterior horn, the occipital lobe was a solid structure, and the calcar avis did not reach deep into the white matter of the lobe, then the forceps would have the shape of a cone with its head at the occipital pole, where cortical fibres would gather like rays equally from all sides. However, now a bulge of the lateral ventricle, which forms the posterior horn, pushes into the forceps from the front, yet not along the axis of the cone, but rather closer to the lower surface. Therefore, the posterior horn is surrounded from all sides by longitudinally running callosal fibres and tears apart the lower part of the forceps. Due to the positioning of the corpus callosum above the ventricle, a major part of the forceps runs anteriorly over the posterior horn (Fig. 3.1). The medial and lateral surfaces of the occipital horn are covered by a thin veil of longitudinally directed callosal fibres (2, 3) with a stronger veil along the inferior surface (4).

The “*large upper part of the forceps*” flexes medially where the posterior horn opens up at the level of the quadrigeminal plate, in order to cross to the other hemisphere. As this part is at the height of the splenium from the very beginning, it is the natural confluence for all forceps fibres. All forceps fibres leave the cortex in a frontal plane, unless they already have entered the callosal layer, and therefore can be traced in their whole length to this point on coronal sections. Direct and unhindered access to the *upper part of the forceps* is only given to fibres from the cuneus and precuneus, as well as fibres from the dorsal and lateral convexity of the hemisphere located above the intraparietal sulcus. These fibres not only join the forceps, but also dig deep into it, before they bend from a frontal plane into a sagittal direction. They thus divide the mass of sagittal fibres of the forceps into a number of tracts and layers. The layering is an expression of the interweaving of all callosal fibres, which continues almost to the medial plane. Thus, callosal fibres from different parts of the occipital lobe lie next to each other. Until their insertion, fibres from the convexity of the hemisphere form a tightly packed, clearly differentiable fibre mass layer in the forceps (5).

On a frontal plane, the remaining callosal fibres originating from the cortex run inferior-superiorly along the occipital horn. Whether these reach their target at the lateral or medial surface of the occipital horn depends upon whether the cortical area they originate from lies lateral or medial on the sagittal plane through the middle of the occipital horn. This plane separates the lingual gyrus from the medial part of the fusiform gyrus at the basal surface. The fibre system originating from the fusiform gyrus – often a tightly packed layer, which is clearly differentiable from the rest of the fibres (6) – climbs vertically and breaks through both sagittal layers by dividing them into three parts. The inner-most part (7) runs at the basal surface of the posterior horn almost horizontal to it and bends slightly upwards, to insert in the yet-to-be-described small part of the forceps. A smaller middle part (8) bends in sagittal direction and strengthens the outer half of the forceps fibres that run sagittally on the inferior [part] of the posterior horn. The lateral largest part (9) runs along the outer surface of the posterior horn, adjacent and lateral to the thin layer of the horn. I shall call all callosal fibres at the outside of the occipital horn “outer forceps layer”. During its course along the outer surface of the posterior horn, this layer is continuously strengthened by fibres originating from the convexity underneath the intraparietal sulcus. These fibres run diagonally from the ventral convexity towards dorsal medial areas. Among them the most ventral fibres are close to a vertical direction. The more dorsal these fibres reach, the more horizontal they run, until they join fibres that cross to the upper part of the forceps directly above the intraparietal sulcus. They form small tracts, visible to the naked eye, that traverse both sagittal layers in the same direction as before and thus divide the latter in even smaller tracts. They then bend upwards in a vertical direction and join the ascending fibres. The whole layer thus becomes thicker as it ascends and bends from a vertical to a sagittal direction at the level of the upper part of the forceps. Also these fibres, like all callosal fibres, do not simply join from below or outside the already existing forceps system; they rather follow the same course of the callosal fibres [originating] from the dorsal cortex, i.e., they penetrate the forceps for a [certain] distance before bending in a sagittal direction.

The fibres of the sagittal veil which are directly adjacent to the lateral surface of the posterior horn (2) traverse diagonally along an anterior – superior [direction] and merge with the dorsal branch of the forceps. In the same way, the thickened bundle bends at the lateral aspect of the inferior occipital horn (8) more anterior and close to the opening of the occipital horn where it runs upwards and diagonally towards the front and then directly upwards to reach the same termination. The lateral sagittal veil shows greater variability. At times it can be clearly seen along the whole length of the occipital horn, in other cases it covers only the posterior part because its fibres bent upwards far more posteriorly and hence strengthen the layer of the vertical ascending fibre. The latter borders directly the ependyma.

The same position is not possible for those forceps fibres originating from the inner part of the fusiform gyrus, the lingual gyrus and the calcar avis at the medial surface of the occipital horn. This is due to the prominent calcar avis that bulges into the occipital horn and hinders a solid development of fibres that do not belong to the calcar avis. The entire forceps fibres originating from the lingual and fusiform gyri that should ascend vertically at this point are running longitudinally along the inferior medial edge of the occipital horn and thereby strengthen the medial half of the longitudinal fibres at the inferior occipital horn. Hence, this forms a cord-like tract, which thickens towards the front (4). Just before the anterior aspect of the calcar avis, directly behind the opening of the occipital horn, this tract has enough room to ascend as “small inner part of the forceps” from within the occipital horn. Once it reaches the roof of the ventricle, this tract bends inwards to join the larger upper part of the forceps and merge with the corpus callosum. The white matter of the fusiform gyrus is adjacent to the above-mentioned fibres that run inferior to the occipital horn (7), whilst the white matter of the lingual gyrus forms a denser layer (10) similar to the one from the dorsal convexity. The fibres from the thin sagittal veil at the inner surface of the occipital horn – the internal forceps layer (3), which are probably joined by callosal fibres originating from the calcar avis, merge anteriorly in the ascending part of the small forceps.

The entire inferior part of the forceps and the sagittal veil at the inner surface of the occipital horn show great variability. Both structures are mutually dependent: If the lower forceps is strongly developed, than the veil at the inner surface will be very fine to the point where it is difficult to appreciate it even at a high magnification and it might only consists of two or three fibre layers. In rare cases however, all of the inferior forceps vanishes and instead forms a tract merging with the veil, which develops as a relatively strong layer that uniformly covers the inner surface of the posterior horn. At times, the inner forceps does not ascend anterior to the calcar avis but ascends more posteriorly in a diagonal direction upwards and forwards.

All forceps fibres are characterised by a strong fibre diameter. The layers of the forceps stain rather dark with haematoxylin, and strongly yellow with picrocarmin.

## 6 Stratum sagittale internum

The stratum sagittale internum wraps around the forceps just as the forceps encases the occipital horn. The fibres of this layer differ from the fibres of the forceps for their smaller axonal diameter. This layer stains very light with haematoxylin, whereas picrocarmin-staining colours this layer in red compared to the surrounding layers. Fibres of this layer originate from the occipital lobe, seemingly from all areas of the occipital cortex, and continue anteriorly into the posterior part of the corona radiata. These fibres form the projection connections, namely the corona radiata of the occipital lobe. To reach their destination, they have to gather at the outer surface of the ventricle. Fibres originating from the occipital pole unify a few millimetres behind the beginning of the forceps as a solid tract that thickens as further fibres join and runs anteriorly along a longitudinal direction. Once these fibres reach the tip of the forceps the tract funnels out and from here onwards encases the forceps from all sides in the shape of an anteriorly widening belt. On sections, fibres of the stratum sagittale internum were not traceable without interruptions along their entire trajectory from the cortex through the white matter. They can only be differentiated with clarity from other fibres, once they form a separate layer. Fibres at the inner surface of the forceps that run longitudinally towards the front (12) as well as fibres originating more anteriorly from the cuneus, precuneus, and lingual gyrus course towards the lateral surface of the forceps – still in the frontal plane – describing an arc around parts of the forceps that course dorsal and ventral to the occipital horn. Once these fibres reach the outside of the occipital horn they bend anteriorly in a longitudinal direction. On coronal sections, the upper parts of these fibres (13) cling to forceps fibres originating from the cuneus and the precuneus. Fibres from the lingual gyrus (14) run in parallel to the above described callosal fibres and course from the lateral to the medial surface in opposite direction from the base of the hemisphere towards the inferior part of the forceps (7).

As a consequence of this arrangement, the part of this layer that lies outside the occipital horn (11) becomes thicker, whereas the part on the inner side becomes finer as the calcar avis progressively penetrates the occipital horn anteriorly, such that it soon becomes only a microscopically visible veil. Eventually, the veil will tear apart just near the callosal bulge to allow the forceps to reach the median surface.

The most inferior fibres of the stratum sagittale internum run almost horizontal along their entire course towards the front. However, the more fibres originate dorso-anteriorly, the sharper their diagonal angle from a dorsal-posterior to an anterio-inferior direction. In the parietal lobe the corona radiata runs eventually vertical on coronal section at the level of the tip of the pulvinar. Thus from here onwards they can be traced along their length on coronal sections.

As already mentioned, lateral to the occipital horn this layer is penetrated by fibres of the forceps that divide it in bundles of equal size. These bundles are visible to the naked eye. Close to the posterior arch of the caudate nucleus the middle part of this layer receives further additions from the yet-to-be-described stratum sagittale externum.

## 7 Stratum sagittale externum

The stratum sagittale externum (15) encloses the just mentioned layer in the same way the stratum sagittale internum covers the forceps. This layer consists mainly of fibres of large axonal diameter. Similar to the forceps, it stains very dark with haematoxylin, yellow with picrocarmin, and is thus clearly differentiated both from the stratum sagittale internum and the surrounding fibres. Whether the numerous fine fibres that cross the sections, which are visible at the level of this layer on coronal sections, are part of it or are just traversing it and strive towards the stratum sagittale internum, I have not been able to confirm with clarity. The latter seems more probable to me. Fibres of this layer originate from the occipital cortex, seemingly from all its areas, and continue towards the temporal cortex except for a small portion. They form the long association tract between these cortices [inferior longitudinal fasciculus]. In order to reach their destination, which is the white matter of the temporal lobe, they all have to gather at the ventral aspect of the ventricle.

Posteriorly the layer appears as a thin belt, which envelopes the stratum sagittale internum equally from all sides and initially describes the same course. These fibres could also not be traced continuously on their way from the cortex to their entrance into the stratum. It seems that these fibres, similar to those of the stratum sagittale internum, do not strive to their collection point like the fibres of the forceps which run vertically from the convexity of the brain on a frontal plane, in a manner similar to the branches of an apple tree to the stem. Rather, they radiate from posterior or diagonally from the cortex, anteriorly towards the ventricle like the branches of a pear tree to the stem. They therefore do not run in parallel to the forceps fibres towards the collecting layers but cross them like clasped fingers.

Fibres from the occipital pole and its neighbouring areas run anteriorly, longitudinal, and parallel to the ventral edge of the ventricle. The fibres underneath the occipital horn maintain their almost horizontal direction whereby they course towards the front and slightly descend in the temporal lobe. For the joining fibres it applies that the more the fibres originate from dorsal-anterior regions the more their direction changes from a dorsal-posterior to an anterior inferior descending direction. Hence, the most anterior fibres of this layer that originate from the convexity where the occipito-parietal sulcus cuts through, meaning from the first transitional gyrus, form an angle of approximately 30° with the most inferior fibres. In post mortem blunt dissections this layer therefore seems like a partially opened fan. At the inner surface of the occipital horn this layer, like the two layers previously described, thins out to a slender veil due to the deep penetration of the calcar avis. This veil behaves anteriorly similar to the thick covering on the lateral surface, in the sense that also here the fibres gradually take a vertical direction.

A consequence of this arrangement is that the stratum sagittale externum progressively tightens towards the base of the brain and forms a “track”, which becomes better defined towards the transition to the temporal lobe. The track consists of a solid foot with bilaterally attached side parts in a rounded right angle. This prominent inferior aspect of the stratum sagittale externum has been termed inferior longitudinal fasciculus by Burdach. Fibres from the cortex form a ridge-like attachment in the middle part of the occipital lobe at several points where callosal fibres penetrate the layer as thick tracts; this is the case dorsally towards the convexity, at the inferior edge of the stratum sagittale externum and towards the lingual gyrus. This attachment becomes very prominent and elongated in the lingual gyrus and has been named by Burdach as the internal basal bundle [inneres Grundbuendel].

Once the stratum sagittale externum reaches the temporal lobe it quickly thins out by sending fibres to the cortex in all directions. When performing dissections, a large part of these fibres from the lateral aspect and the foot of this layer can be followed into the first temporal gyrus. A smaller part reaches the second temporal gyrus, and the remaining fibres become insignificant and reach towards the temporal pole where they inseparably merge with the white matter of the temporal lobe and continue anteriorly. The most anterior fibres of this layer terminate in the pole of its lobe. In the anterior aspect of occipital lobe and the precuneus, sparse fibres descend diagonally from the medial surface of the occipital horn and after joining the cingulum they bend around the splenium and then continue with it – and in healthy brain they are inseparable from it-towards the temporal lobe.

Within the occipital lobe the stratum sagittale externum is divided into small, equal bundles by penetrating forceps fibres just like for the stratum sagittale internum.

A small amount of fibres of the stratum sagittale externum that lies lateral to the occipital horn does not reach the temporal lobe; instead the medial part of this subdivision segregates into numerous small bundles that are visible to the naked eye. These bundles are entangled like ropes and penetrate the stratum sagittale internum. They are differentiable within the latter due to the larger axonal diameter and their dark staining with haematoxylin. Both structures jointly reach the foot of the corona radiata.

## 8 Stratum proprium corticis

The majority of white matter between the stratum sagittale externum and the cortex has almost the same diameter in all directions as the three inner layers together. This white matter consists mainly of small association fibres, which originate and terminate within the occipital lobe. It is penetrated by long ranging fibres originating from the cortex and thence merging with the three inner layers. The short fibres mainly run in the frontal [coronal] plane, and thus interconnect dorsal and ventral or medial and lateral regions and only rarely do they interconnect directly adjacent cortical areas.

Three such fibrous tracts originate from the dorsal aspect of the cortical regions above the calcar avis.

The most important of these fibres is the ***stratum calcarinum*** (16), which consists of fibres that circumvent the calcar avis in its full extension, and the longest of which connect the cuneus to the lingual gyrus. In the white matter strips of the three above-mentioned vertical short gyri, which are placed on the insular ground of the calcarine fissure, this layer thickens into three strong bundles. Among these bundles the most anterior is rather prominent and partially reaches the base of the hemisphere. As a result of this filling of the “gyral comb”, the respective sulci do not appear at the bed of the calcar avis on the inner surface of the occipital horn. Anteriorly this layer reaches beyond the connection point of the calcarine fissure and the occipito-parietal sulcus into the temporal lobe and envelopes in a similar fashion the continuation of the calcarine fissure by connecting the cortex of the uncinate gyrus with the lingual gyrus.

The second layer originates from the dorsal cortical region of the calcar avis, the stratum cunei transversum (17). In contrast to the stratum calcarinum this layer only exists in the region of the cuneus and does not extend beyond the confluence of the calcarine fissure in the occipito-parietal sulcus. The fibres of this layer originate together with those of the stratum calcarinum and initially run parallel with them over the dorsal calcar avis from medial to lateral. However, rather than bending downwards after the calcar avis they continue in the same direction above the dorsal part of the stratum sagittale externum and bend downwards on the other side of the latter to follow its lateral surface. On coronal sections cut through the posterior half of the occipital horn, this layer can be seen to reach the inferior border of the stratum sagittale externum: the more anterior the less these fibres reach inferiorly and the thinner the whole layer becomes until they eventually vanish in the region of the anterior occipital sulcus. Thus far it has not been possible to trace these fibres in isolation upon their exit from this layer along their trajectory through the stratum proprium convexitatis towards the cortex. They potentially reach the cortex of the whole convex region and part of the inferior occipital cortex, thus forming an association pathway between the cuneus and the convexity. It seems that these fibres have a straight direction only in their posterior and medial part along a medial to lateral course, however, more laterally and anteriorly, they bend out of the frontal plane and than continue diagonally along a posterior medial to anterior lateral direction. Thus the longest fibres of this layer might reach the dorsal parietal lobe and possibly the angular gyrus.

A subtler, yet at times quite prominent, analogous layer originates from the lingual gyrus and continues inferiorly around the stratum sagittale externum. In an ideal situation one can appreciate a fifth layer, which envelopes the latter stratum from all sides in the posterior frontal plane, where the occipital horn still is slit-like, between the stratum proprium cortices and the stratum sagittale externum.

Both layers, namely the stratum calcarinum and stratum cunei transversum, stain rather dark with haematoxylin yet less than the stratum sagittale externum. Therefore, they can be clearly differentiated from it and from the rest of the white matter.

The third layer, namely the stratum proprium cunei (18), which originates from the upper edge of the calcar avis, ascends perpendicular to the dorsal hemispheric margin and encapsulates the fissure of the cuneus that runs parallel to the calcarine fissure.

These three layers originating from the cuneus, seemingly form a joint system of short association fibres, which interconnect the cortex of the cuneus with the entire occipital cortex.

Similar to the region near the calcar avis, the space between the cortex and the stratum sagittale externum lateral to the occipital horn is filled by a stratum verticale convexitatis, which runs vertically in a dorso-ventral direction. Each of the three sagittal occipital sulci is enveloped by a system of gutter-like fibres [U-shaped fibres], which connect the gyri above and below the sulci; stratum proprium sulci occipitalis I s. interparietalis (19), str. Pr. S.o. II (20), str. Pr. S.o. III (21). A fourth system, the stratum proprium sulci collateralis (22), connects the lingual gyrus with the fusiform gyrus at the base of the brain. The more medial one reaches from the lateral aspect of the brain the longer the vertical fibres become. The fibres that follow the superficial strata propria of the sulci hurdle only one gyrus.

The deepest fibres directly abutting the stratum sagittale externum or stratum transversum cunei project along the whole height of the lobe and interconnect as stratum profundum convexitatis (23) the dorsal and ventral margins of the hemisphere.

This prominent vertical fibre system, the stratum proprium [verticale] convexitatis, is consistent across the whole posterior part of the cerebrum. Anteriorly it extents beyond the occipital lobe and gradually becomes thinner before its sharp boundary in the white matter strip between the postcentral and the intraparietal sulci within the parietal lobe. Further inferior the most anterior vertical fibres encase the insertion point of the interparietal sulcus with the postcentral sulcus. They than reach the supramarginal gyrus from where they course anterior in the depth to join the association fibres of the insula that ascend from the operculum. In the temporal lobe, the most anterior fibres descend from the inferior aspect of the angular gyrus towards the second [middle] temporal gyrus and form the floor of the superior temporal sulcus, which at this point is often interrupted by a small vertical gyrus.

The stratum verticale convexitatis is also strongly developed in the monkey and has been described as fasciculus occipitalis perpendicularis by Wernicke (as previously cited, p. 23).

Similar to the sagittal sulci, both vertical sulci, namely the anterior occipital sulcus and the ascending branch of the superior temporal sulcus, are encapsulated by a very thin groove of longitudinally directed short association fibres.

In the precuneus, the layer of fibres adjacent to the cortex, namely the stratum proprium praecunei, also has a vertical direction and encapsulates the posterior elongation of sulcus calloso-marginalis in dorso-ventral direction. More medially located fibres bend anteriorly at their inferior terminations and join the dorsal part of the cingulum whose detailed description is yet outstanding. The deeper these fibres run, the farther anterior they penetrate the cortex of the gyrus fornicatus [the upper limb is the cingulate gyrus and the lower limb is the parahippocampal gyrus]. A third layer of vertically directed fibres is formed by the fibres previously described as belonging to the anterior medial part of the stratum sagittale externum and joining the descending part of the ventral cingulum reaching the temporal lobe. The second mentioned layer belongs to the anterior part of the precuneus, whereas the third belongs to its posterior part. Subsequently, fibres of the corona radiata follow that ascend towards the hemispheric margin.

In the anterior region of the occipital lobe and at the transition to the parietal lobe, where the stratum cunei transversum terminates, it remains a white matter system surrounded by the stratum proprium praecunei medially, the stratum verticale convexitatis laterally, and the stratum sagittale externum ventrally. This system abuts the superior part of the stratum sagittale externum like a roof ridge and consists mainly of fibres that run in a longitudinal cranio-caudal direction. This fibre system is only clearly visible on fresh coronal sections of a brain hardened in the Müller solution. It appears as a brighter area, which abuts the stratum sagittale externum like a cap and is distinguishable from the deep dark transverse cut of the latter, whilst it becomes gradually indistinguishable towards the dorsal and lateral white matter of the stratum proprium corticis. It seems that this cap does not represent an association pathway in its own right but its curious appearance is rather owed to the fibres that pierce through here and then adhere to reach into the stratum sagittale externum and internum. It is possible that this area is also in relation to the most anteriorly bending fibres of the stratum cunei transversum. This is not noticeable in stained sections of a healthy brain.^2^

A similar smaller fibre system is present between the inferior part of the stratum sagittale externum and the stratum proprium sulci collateralis. A third system, at times in continuity with the just mentioned system, is found in the lingual gyrus close to the cortex of the calcar avis.

All these layers within the stratum proprium corticis, except the first mentioned stratum calcarinum and stratum cunei transversum, stain proportionally weak with haematoxylin.

With regards to the relation of size and form of all these white matter layers a look at the attached photographs will allow a better overview than any thorough description. Here, only the following will be mentioned, as it seems important with regards to pathology.

As mentioned above, the incision of the sulci into the white matter only affects the configuration of the outer most layer, the stratum proprium cortices, but only marginally the shape of the three inner layers (not even the stratum transversum cunei). Only the three layers of the calcar avis thin out to veil-like coverings. The medial occipito-temporal sulcus causes a concave invagination of the lower margin of the stratum sagittale externum; whilst the thickness of the stratum proprium corticis depends on the proximity of the cortical sulci to the stratum sagittale externum. At the medial surface of the brain this effect is visible in the thickening of the three above described gyri breves calcaris avis that form the stratum calcarinum. At the outer surface, the stratum proprium is pushed together by both vertical sulci of the occipital lobe, less so by the anterior occipital sulcus but [even] more by the ascending branch of the superior temporal sulcus. The stratum verticale convexitatis is especially thinned by the cortex of the most posterior protrusion of the Sylvian fissure. The thinner the outer layer, the easier a lesion that is originating from the cortex can reach the inner layers. A lesion progression from the cortex is thus easiest at the posterior end of the Sylvian fissure and underneath the second parallel sulcus, hence the region of the inferior parietal lobe.

Consequently, a superficial softening within this region can, depending on its depth, isolate the stratum sagittale externum or damage both the stratum sagittale externum and the stratum internum. This can cause transcortical syndromes such as optic aphasia (Freund) or apperceptive soul blindness [associative agnosia] (Lissauer) due to an interruption of the connections between visual and auditory centres. When the disconnection is present in association with a subcortical disturbance this causes hemianopsia. On the other hand, a lesion originating from the ependyma of the occipital horn may disrupts, apart from a small part of ascending forceps fibres at the lateral surface of the occipital horn, the entire stratum sagittale internum in its function and thus cause pure subcortical hemianopsia without cortical or transcortical syndromes.

Additionally, two other tracts remain to be described, which are in close spatial relationship to the occipital lobe, especially to the stratum verticale convexitatis and stratum calcarinum. They do, however, not continue as part of the lobar white matter and are not in relation with its cortex. These are the arching fibres and the cingulum (Burdach). The arching fibres (respectively the fasciculus arcuatus or dorsal longitudinal fibres or longitudinalis superior) correspond to the stratum verticale convexitatis of the occipital brain in their course through the anterior parts of the brain. It is located in the depth of the dorsal gyrus of the Sylvian fissure, namely the operculum; its fibres extend dorsally approximately over half the height of the convexity. It consists of short association fibres, which connect neighbouring gyri with each other. In deeper layers, these association fibres bypass one gyrus at most. I doubt it contains long association fibres, which connect distant cortical areas. The deepest fibres of this tract running in the bed of the dorsal sulcus of the insula seem to have a special function. The direction of these fibres is always perpendicular to the direction of the corona radiata. In the region of the central gyrus and the dorsal part of the marginal gyrus these fibres run horizontally. At the transition point from the parietal lobe to the temporal lobe it bends downwards and joins the stratum verticale convexitatis whose anterior projections are identical with these fibres. Haematoxylin stains the arcuate fasciculus relatively light.

Along the medial aspect of the hemisphere the cingulum runs with a trajectory that is similar to the arcuate fasciculus. It originates underneath the callosal rostrum in the most posterior aspect of the inferior surface of the frontal lobe [subcallosal gyrus] as a thin wide layer that is inferiorly abutting to the corpus callosum. Initially, the fibres continue diagonally upwards and then form a bundle that bends dorsally around the genu and horizontally abutting to the corpus callosum directly underneath the cingulate gyrus. The cingulum runs along the entire length of the corpus callosum before it bends around the splenium and projects to the parahippocampal gyrus in the temporal lobe. When disregarding its frontal lobe trajectory, the cingulum can be segregated into a dorsal part, a descending part, and a ventral part. The cingulum consists of numerous small fibres that only stain lightly with haematoxylin and a compact tract of long dark staining fibres. The dorsal part of the cingulum includes the above-mentioned fibres that connect the cortex of the precuneus with the cingulate gyrus. The descending part separates the splenium from the anterior fibres of the stratum calcarinum from which it is distinguished by its dark haematoxylin staining. The inferior part is joined by anterior medial fibres of the stratum sagittale externum, which originate from the precuneus.

The above-mentioned tracts and layers can be visualised as detailed below by using the methods described above. The stratum verticale convexitatis can be easily demonstrated with blunt dissections where the cortex is broken off with a scalpel and the fibres are then removed in bundles using tweezers. This procedure permits to appreciate the sharp anterior border of the layer and the anterior tracts of the temporal lobe that come together with the descending fibres of the arcuate fasciculus. If prepared carefully it is possible to visualise fibres that run through this layer and project medially along the white matter strip of the gyri. On fresh sections through a brain hardened in Müller solution, this layer appears light green on coronal or sagittal cuts and dark green on axial cuts. The stratum calcarinum and proprium sulci collateralis can be equally dissected, although, with more difficulty and only if they are well developed. They visualise similarly on fresh sections. I was not able to demonstrate the stratum transversum cunei with blunt dissections. On fresh sections of a well-hardened brain, however, this can be distinguished from stratum verticale convexitatis by its darker colour. The stratum proprium cunei is especially marked as a green-black transection on axial cuts of fresh sections. All these layers of the stratum proprium corticis are relatively strongly de-stained when using Pal staining. The strongest de-staining occurs for the stratum profundum convexitatis and a lesser effect is seen for the strata propria of the sulci; the stratum calcarinum and stratum cunei transversum remain dark blue, though still lighter than the stratum sagittale externum.

Blunt dissection is particularly good for the lateral surface of the stratum sagittale externum when the fibres of the cortex, which are perpendicular to them, are fully removed. The foot of the stratum sagittale externum is best demonstrated by dissecting from medial aspects where it is the basal bundle of Burdach and reaches close to the cortex of the lingual gyrus. When the calcar avis is reached the layer becomes too thin for further dissection. More anterior, however, the fibres from the precuneus that join the inferior part of the cingulum are well demonstrated. On fresh sections, this layer appears black on frontal sections and lighter on parietal sections especially in the dorsal parts due to the majority of descending fibres. It is distinguished from the stratum sagittale internum as well as its abutting cap by a different shade of stain. On axial cuts, however, the layer is light green and only on more dorsal cuts in anterior regions does it appear darker. The inner border between stratum sagittale externum and internum cannot be demonstrated with blunt dissection as the fibres of both layers have the same direction over long distances. The stratum sagittale externum is clearly distinguishable in all its parts from surrounding fibres when using Pal-stained sections – the stronger the de-staining of the section, the better the distinction. This stain is adequate for this layer. It stains strongly dark blue and can be followed under the microscope into its fine branches at the medial aspect of the occipital horn.

As mentioned above, the stratum sagittale internum cannot be clearly visualised by dissections beginning from the convexity, however, when starting from the medial surface its visualisation is possible when removing all callosal fibres. On fresh sections, this layer is distinguished from the stratum sagittale externum lateral to the occipital horn by a different shade of colour. Fibres that run transversely inferior and dorsal to the occipital horn are white on coronal cuts. When using Pal staining this layer stains only lightly and gains a brownish shade from which the dark blue callosal fibres, that traverse this layer, can be clearly differentiated. Picrocarmin stains this layer reddish compared to the surrounding structures and shows its nuclei in a row along the penetrating callosal fibres.

The forceps is nicely shown in its entirety with blunt dissection; with the obvious exception of single fibres that penetrate the surrounding layers. On fresh sections the fibres that run underneath and lateral to the occipital horn towards occipital and dorsal regions penetrate the strata sagittalia. These fibres appear whitish on frontal cuts everything else appears black-green. On axial sections the association and commissural fibres are whitish and projection fibres are black-green. The Pal method stains these layers of the forceps almost as dark as the stratum sagittale externum.

It is easy to reveal the arcuate fasciculus with blunt dissection. On fresh coronal cuts, it appears as a dark slim ellipsoid – adjacent to the corona radiate – that sends a branch into the operculum; it completely disappears behind the Sylvian fissure. When using Pal staining, the arcuate [fasciculus] is not distinctly visible anywhere. The only change that becomes evident on coronal sections is that the region anterior to the caudal end of the Sylvian fissure where the arcuate is passing through is slightly lighter than the surrounding area after strong de-staining.

Blunt dissection nicely demonstrates the cingulum along its entire length including both its short and long fibres. On fresh coronal cuts, the long fibres appear as a black-green field that is abutting to the callosum and penetrates the cingulate gyrus. Behind the splenium it appears as a white–green thin cord with a dorso-ventral direction. On fresh axial cuts, it has exactly opposite colours. In the temporal lobe the cingulum disappears as an independent area. The Pal method stains its short fibres light, the long fibres dark blue, however, not as dark as the stratum sagittale externum.

Burdach (1822) has dissected the “inferior longitudinal fasciculus” with a branch towards the frontal pole. This is only possible, when coming from the stratum sagittale externum in the anterior temporal lobe to the posterior extension of the uncinate fasciculus, which covers the latter and connects the temporal pole to the orbital frontal lobe. Such a trajectory can be artificially produced with blunt dissection. The longest fibres of the uncinate fasciculus originate at the inferior lateral margin of the hemisphere, where the shortest fibres of the stratum sagittale externum, coming from posteriorly, terminate. This might therefore be seen as the area that best defines the border between the occipital and the temporal lobe. However, this could evoke the false impression of an uninterrupted trajectory of fibres through both bundles. On histological cuts it is immediately evident that this is just a deception, as both layers remain clearly distinct from each other. On coronal sections through the temporal lobe, the stratum sagittale externum becomes a slim horizontal dark line and disappears fully to the naked eye long before it reaches the temporal pole.

Meynert (as cited, page 41) believes that it is possible to follow the fibres of the anterior commissure into the occipital pole using blunt dissection. I was not able to replicate this. I could only follow fibre bundles of the anterior commissure up to the inferior margin of the cortex of the temporal lobe and I am convinced that the majority of these fibres end here (see also Wenicke as cited, page 86). A margin of error is given here, as fibres of the anterior commissure cross those of the stratum sagittale externum diagonally, thus permitting one to easily get from one fibre layer into the other during dissection. Anterior commissure fibres can not be followed beyond the temporal lobe neither on fresh nor on histological coronal cuts.

Onufrowicz (1887) and Kaufmann (1887; 1888) have studied brains with congenital agenesis of the corpus callosum in which they found that the “tapetum” of the temporal and occipital lobes was present. Both authors could follow the tapetum anteriorly as a thick longitudinal fibre bundle, which they referred to as superior longitudinal fasciculus or arching bundle [Bogenbündel] of Burdach and believed it to be visible due to the absence of the corpus callosum. They thus inferred that the tapetum is not part of the corpus callosum, but rather the postero-inferior part of a large fronto-occipital fasciculus. This tract has thence been referred to as “fasciculus fronto-occipitalis” [superior fronto-occipital fasciculus] in textbooks by Obersteiner and Edinger.

I take the liberty to suggest here, that in order to avoid confusion already known structures of the brain should be referred to using the terminology introduced by Burdach until a full review of anatomical terms has been conducted. Burdach only refers to that part of the corpus callosum as tapetum, which runs towards the base of the brain, lateral to the lateral ventricle, and then continues anteriorly into the temporal lobe. Burdach refers to all remaining projections from the callosum to the occipital lobe as “forceps”. In more recent publications, even the fibres ascending at the lateral surface of the occipital horn and merging with the dorsal forceps are called tapetum. Both these layers correspond to each other and merge into each other at the opening of the occipital horn; yet, they can be differentiated from each other. The posterior fibres, which bend anteriorly and thus reach the temporal lobe, are the terminations of the tapetum. Fibres, that follow afterwards, of which the first descend straight [while] the later run towards the occipital lobe for a short distance in the dorsal forceps before descending, are part of forceps and constitute the anterior part of this layer that ascends towards the forceps along the lateral surface of the occipital horn. The border between both layers lies just behind the posterior arch of the caudate nucleus.

To my believe, both above-mentioned authors have mistaken the superior longitudinal fasciculus or arcuate fasciculus located close to the lateral convexity with the cingulum, which is located at the medial surface and separated from the arcuate by the corona radiata and the stratum sagittale externum. Owing to the absence of the callosum, the cingulum is positioned more inferior. The arcuate fasciculus^3^ was not only hinted at by Burdach, as suggested by Onufrowicz, but was distinctly described by him. It is indeed easy to demonstrate this bundle in the healthy brain using blunt dissection or fresh cross-sections.

According to the description and the figures from both publications it can only be inrefered that these fibres belong to the dorsal part of the cingulum and posteriorly merge with ascending fibres of the forceps. Though I have looked with outmost care, I was not able to follow any fibres from the dorsal part of the cingulum to the occipital lobe. The cingulate fibres are limited to the cingulate gyrus [Randgyrus des Balkens]. Unless they terminate within the anterior part of the precuneus or the descending part of cingulate gyrus, these fibres run in an arch around the splenium and reach the temporal lobe. Likewise, on fresh and stained sections it is impossible to demonstrate that cingulate fibres, which are clearly distinct everywhere, reach the occipital lobe.

Owing to Mr. Kaufmann's courtesy I was able to re-examine his anatomical preparations. I hereby arrived at the conclusion that this is not indeed an acallosal brain. The fibres of the corpus callosum are all present; they merely do not transverse to the contralateral hemisphere but rather remain in the same hemisphere and run anterior-posteriorly. Thereby producing a fronto-occipital bundle in the ‘acallosal brain’ that is completely absent in the healthy brain.

Such heterotopy of the corpus callosum is of no importance for the study of the normal brain anatomy, yet can be important for the study of its development.

More recently, Mingazzini (1890) indeed described a brain with complete callosal agenesis where the ascending forceps fibres and tapetum were also absent.

With regards to Hamilton's repetition of Foville's belief that the corpus is a cross-over of both internal capsules, the following is the case in the occipital lobe: callosal and projection fibres are clearly distinguishable from each other. Fibres from the posterior part of the foot of the corona radiata run ipsilateral towards the occipital lobe within the stratum sagittale internum and, to a smaller extent, within the stratum sagittale externum. [Also] there is no evidence that the forceps forms a commissure of both occipital lobes. For the time being, we cannot even speculate on the continuation of fibres after they come from the forceps on one side and traverse to the other hemisphere. They might reach totally different, anterior cortical regions or even reach the internal capsule. Both methods, namely blunt dissection and histology, fail to answer this question. In the future, this question might be addressed with unilateral lesion studies.

I believe that the widely accepted notion that the function of the corpus is to connect homotopical cortical regions (see Meynert as cited p. 41; Wernicke as cited p. 23) is wrong or at least incomplete. There is no evidence for this *a priori* opinion. Against this opinion stands the fact that callosal fibres entangle prior to reaching the midline. Most likely, fibres from certain areas of one hemisphere disperse in different directions after crossing the midline. There is no reason to assume that these fibres, instead of reaching their destination on the shortest possible way like all other fibres, reach the midline totally arbitrarily; and that they then so radically change their position that they come to lie smoothly in the same order next to each other as they did at the beginning.

The argument that Hamilton uses against previous scientists, especially Meynert, namely that it is impossible to follow a single fibre from one area of the cortex to the homologous area in the other hemisphere, also stands against Hamilton himself. It is equally not possible to follow a single fibre from the cortex to the internal capsule of the other hemisphere.

Generally, I agree with Schnopfhagen's (1891) interpretation of the corpus callosum as a “bed of association fibres, which connects structurally and functionally totally different regions of the hemispheres”. It is beyond my judgment, if a minority of callosal fibres might reach the internal capsule in the frontal lobe as postulated by Hamilton. Schnopfhagen contested this opinion. In the posterior regions of the brain it seems that no callosal fibres enter the foot of the corona radiata.

Physiology postulates at least two tracts in the forceps. Assuming that the regions for focal vision are presented in the occipital lobes, a commissural tract within the forceps is to be expected, which connects the visual cortices. Additionally, there has to be a direct connection from the right occipital lobe to the left temporal lobe, which allows the naming of objects seen in the left visual field and which has to be disrupted in cases of optic aphasia of Freund. This tract is probably found within the forceps on the right side and within the tapetum on the left.

I ought to comment on a statement by Schnopfhagen on the straight occipital bundle of Wernicke. Schnopfhagen says (p. 102): “Wernicke describes a “straight occipital tract”, a fibre bundle running from dorsal to inferior, which connects the second temporal gyrus (namely the Pli courbe, the dorsal part that is neighbouring onto the precuneus) with the fusiform gyrus [Spindelwindung]. A drawing of this tract, based on an axial cut through a monkey brain, is available in his book on brain pathologies (Fig. 19 *ff*). It seems to me beyond doubt that this “straight occipital bundle” is nothing but a plaited area at the convex lateral surface of the occipital horn.”

It seems to me rather brave to reach an opinion “beyond doubt” based on schematic drawings of a third person, such as Wernicke's figure 19, from which a third party gained its assumptions. The “straight occipital bundle” is a collection of association fibres, which are evident in the monkey brain on horizontal cuts and especially on sagittal cuts where they appear as sagittally cut fibres. A triangular plaited region on axial sections, which is distinguishable from the rest of the fibre mass as a base of a gyrus at the convex lateral surface of the wall of the occipital horn exists neither in the monkey nor in adult human brain. In the human brain, the association fibres of the stratum profundum convexitatis are so prominent that individual fibres from the callosum, the corona radiata or long association fibres running towards inner layers fully disappear within this system.

The following conclusion do actually not belong here but are rather destined for the end of the work dedicated to the entire white matter anatomy of the cerebrum. Meynert's theory about the development of psychiatric activity is based upon the anatomical assumption that each part of the cortex is in direct anatomical connection to each other, such that between any two random cortical regions association tracts can be carved out (Meynert, p. 138). My research thus far does not support such an assumption as a general rule. The occipital lobe has only one long association tract, namely the stratum sagittale externum that connects to the temporal lobe [inferior longitudinal fasciculus]. Possibly, there might also be some minor connection via the anterior fibres of stratum transversum cunei between the cuneus and the posterior part of the parietal lobe. Apart from these there is no evident connection with the parietal or frontal lobes (neither their convexity nor their medial surface), which would be comparable to the dimensions of the connection reaching the temporal lobe. Likewise, apart from the temporal lobe, there is no significant long association tract between two physiologically distant brain regions. Provable connections are limited to the vicinity and even the longest of these stay within the borders of each lobe. Any long [interlobar] fibres would therefore have to be relatively few and isolated.

However, this is rather different for the temporal lobe. The temporal lobe has a strong connection with the occipital lobe via the stratum sagittale externum. An important, though less prominent connection to the frontal lobe is via the uncinate fasciculus. The cingulum connects the temporal lobe to the precuneus, paracentral lobe and the part of cingulate gyrus that lies above the callosum. The cingulate fibres might even reach the frontal lobe. The temporal lobe is in connection with the parietal lobe via the posterior part of the arcuate fasciculus or the anterior fibres of the stratum verticale convexitatis. Additionally, it is the only lobe to have commissural fibres, meaning that for the anterior commissure is true what is not the case for the callosum: fibres in both hemispheres run in the same way without crossing or entangling.

In comparison to these very prominent associations, the corona radiata of the temporal lobe is relatively insignificant. Apart from the fornix, which connects to the mammillary bodies and possibly also to a cortical area, only a small amount of fibres enters the internal capsule.

This arrangement is possibly the anatomical expression of the psychological fact that language is of utmost importance for human thought process. Words and sounds have direct anatomical connections with all primary cortical areas for sensory perception, whereas those areas themselves are only indirectly connected via the speech centre. All separate parts of thought, which eventually are composed from the memory of various sensual perceptions, are in essence connected by the medium word, which expresses the thought.

Thus the anatomical study of the brain makes one understand the incredible power the word has for human beings, in their every day life, but also in hallucinations of the mentally ill, and the confabulations of the hypnotised. This physiological arrangement of the thinking organ might be the reason for the phenomenon that a congenital blind person is able to develop all higher cognitive functions despite the lack of the most noble of senses, whilst deaf-mute people only on rare occasions can rise above the level of an animal.

## 9 Annotations to the enclosed photographs [1–8]

The enclosed photographs are taken from specimens stained with the Pal method and are represented according to their natural size. Vertical lines 1–6 in Figs. 1 and 2 represent the approximate level of each cut.

1. The cut is located approximately 25 mm anterior to the occipital pole. Apart from atypical sulci, this cut shows the transection of the beginning of the three occipital sulci (as above I, II, III) on the lateral convexity and the collateral sulcus on the inferior surface (coll.). The medial aspect shows the calcarine fissure (f.c.) with its prominent dorsal and inferior gyri. These are evident even with strong de-staining of the specimen due to the white matter strips running within the cortex as well as the sagittal running sulcus of the cuneus (cu.). The posterior horn is not yet visible on this section. Also the callosal fibres have not yet united to form a distinct layer. The medial aspect of the white matter offers only two layers that are concentric and form a triangle with the dorsal tip and a ventral base. This triangle is roughly equal to the size of the calcar avis. The light layer in the middle is the stratum sagittale internum (1), the lateral darker layer the stratum sagittale externum (2). The white matter towards the cortex, which is the white matter of the occipital lobe, the stratum profundum convexitas (10) is stained relatively light, the strata propria of the cuneus (5) of the three occipital sulci (6,7,8) and the sulcus collateralis are stained slightly darker. Even darker, yet lighter than the stratum sagittale externum, is the stratum calcarinum (4), which is located between the latter and the cortex of the calcar avis. The same shade is evident for the stratum transversum cunei (3), which jointly originates with the latter fibres from the cuneus and runs dorsal and lateral to the stratum sagittale externum. This [3] can be followed as a slim grey strip on the lateral aspect of the stratum sagittale externum to the inferior lateral margin. Hence, this plane shows a total of five encapsulated layers, which differ in their staining intensity and can therefore be separated.
2. This cut is located approximately 1 mm anterior to the previous one. This section shows the forceps as a dark stipe that is thin dorsally and widens inferiorly. It is located in the middle of the homogenous stratum sagittale internum as described on the previous section.
3. This cut is located approximately 5–10 mm anterior to the previous one and approximately 30 mm away from the occipital pole. On the medial margin this section demonstrates the calcarine fissure (f.c.) underneath a deep sulcus of the cuneus. Compared to the previous section, the foundation of this fissure is deformed from a wide base to a thin ditch. On the dorsal cortex of the calcar avis, as well as on the ventral cortex, a secondary sulcal development is evident. The inferior margin of this section shows the section through the well-developed collateral sulcus (coll.). On the lateral convexity there are five evident transverse sulci. The four inferior ones of these equal the three longitudinal occipital sulci with the dorsal one forking into two branches (s.o. I–III). The calcar avis is underdeveloped which is compensated for by a dominant protrusion of the occipito-parietal sulcus into the white matter of the occipital lobe. This sulcus merges with the main sulcus (o.), which infringes from the convexity and then runs posterior. On this section the posterior horn can already be appreciated as a triangle with a small base, the medial and inferior borders concave and the lateral border convex (v.). The indentations of the medial and inferior borders are necessitated by the intrusion of the calcarine fissure and the collateral sulcus. Amongst the medial white matter layers the forceps (1) has a prominent dorsal part and a less prominent spoon-tongue-shaped inferior part that is reaching into the lingual gyrus. Medially to the occipital horn the single layers cannot be differentiated due to the overall dark staining on the section. The bright area at the inferior half of the forceps fibres located lateral to the occipital horn is not a genuine appearance but originates from an error during the cutting of this section. The reason for this is the presence of a vessel in the ependymal of the occipital horn, which damaged some fibres along several sections. The stratum sagittale internum (2) can be appreciated as a bright area dorsal, lateral and inferior to the forceps. It then continues as a beak-shaped protrusion into the lingual gyrus. Medial to the occipital horn the layer has become too thin for examination with the naked eye. The same applies to the dark stained stratum sagittale externum (3). The inferior aspect of it [3] is thinned and bend due to the collateral sulcus, whilst the intruding sulci only cause a slight curvature on the lateral aspect. It already becomes evident on this section that the infero-lateral aspect of this layer is more dominant than other parts.

The stratum profundum of the convexity (10) is lightly stained whilst the layers of the sulci (6, 7, 8, 9) are easily differentiated due to their darker stain as we have already seen on previous sections. Exceptionally dark – nearly comparable to the stratum sagittale externum – are the stratum calcarinum (5) and the stratum transversum cunei (4) whose common origin in the cuneus is clearly visible. At the lateral dorsal border of the cortex, the stratum transversum cunei forms a helm-shaped cap, which is formed by its dorsally projecting fibres. It thence continues as a slim stripe at the lateral surface of the latter with the result that it nearly reaches its latero-inferior border.

4. This cut is located approximately 30 mm anterior to the previous one and approximately 60 mm away from the occipital pole. The majority of this section is not located in the occipital lobe anymore. The lateral aspect shows the dorsal parietal lobe (I) and underneath it the inferior part of the angular gyrus (II). The medial aspect shows the precuneus (VIII) and – as the most anterior part of the occipital lobe– the anterior termination of the cuneus, which is thinning to remain as slim stripe (VI). The main fissures of the surface anatomy that can be appreciated here are the intraparietal sulcus (i) and at the inferior margin of the hemisphere the third occipital sulcus. The latter might already be referred to as the third temporal sulcus here (s.o.III). The collateral fissure (coll.) is again visible adjacent to the inferior part of the stratum sagittale externum. On the medial aspect the fissure calcarina (f.c.) and the occipito-parietalis sulcus (o.) are abutting just after they have merged. The cross-section of the precuneus shows the posterior elongation of the calloso-marginal sulcus (cm).

The occipital horn in this particular specimen is rather wide in its anterior half. In comparison to the previous section, it gained in width and formed a prominent dorsal surface, which is protruding convex into the ventricle dorsally due to the protrusion of the dorsal part of the forceps. The dorsal part of the forceps (1) gained significantly in size and continues at the lateral surface of the occipital horn (2) into the vertically ascending fibres. These fibres appear as longitudinally cut under the microscope (compare figure 3 and 9). The forceps fibres underneath the occipital horn are cut longitudinally where they reach for the stratum sagittale internum and are cut transverse where they are close to the ventricle (Fig 3.7). The inferior part of the forceps (4) is still located at the inferior margin of the occipital horn. The connection between this and the dorsal part is formed by a thin layer of fibres that are cut transversely and that run along the inner surface of the occipital horn, namely the medial forceps layer (3). The stratum sagittale internum (5) disappeared where the calvar avis is penetrating the white matter and is not visible in this specimen under the microscope. The part of it that is located lateral to the occipital horn is formed by transversely cut fibres, whilst its fibres dorsal and inferior to the forceps are cut longitudinally and constitute the addition to this layer that comes from the cortex of the medial occipital lobe. The beak-like extension of the stratum into the gyrus lingualis, which was already present on the previous section, can still be visualised here. The beak appears as a transversally cut fibre bundle under the microscope. The stratum sagittale externum (6) is similar to the internum in its shape. Its inferior part is further thinned and bend due to the collateral sulcus. On the lateral aspect it is already visible to the naked eye that the layer is disappearing due to the various penetrations of thin bundles of fibres designated to reach the forceps. In the inferior part the fibres are transversely cut whilst in the dorsal part they are cut aslant. When comparing this section to the previous one, the formation of bundles from forceps fibres is evident in the region between the stratum sagittale internum and the externum. The strata priopria of the interparietal sulcus (10) and the collateral sulcus (12) are clearly distinct from deep layers of the cortex (9), as the latter is stained lighter. The dorsal part of the stratum sagittale externum is covered by a cap that appears darker compared to the surrounding fibres. These lighter fibres are the anterior remnant of the stratum transversum cunei, which will disappear more interiorly together with the cuneus.

5. (Enlargement ^9^/_8_) This cut is located approximately 5 mm anterior to the previous, approximately 65 mm away from the occipital pole, and only few millimetre before the posterior part of the corpus callosum. This section therefore covers entirely the parietal lobe. The remnant of the cuneus that was still visible on the previous section has now disappeared and made room for the descending part of the cingulate gyrus (VIII). Dorsal to this the precuneus (IX) is cut along its largest diameter. With regards to the fissures on the convexity, the interparietal sulcus (i) is cut diagonally and the ascending branch of the parallel sulcus (e.) is cut longitudinally. Underneath the latter one can appreciate the transversely cut second and third temporal sulcus. On the basal aspect one can see the collateral sulcus again the indents the stratum externum and on the border to the inferior medial aspect the anterior and shared part of the calcarine fissure with the occipito-parietal sulcus (f.c.).

The basal aspect is reduced in size in relation to the other two as well as in its absolute diameter and its direction got closer to the medial surface, meaning it is more vertical. The convexity on the other hand is approaching the hemispheric midline inferior just as it always has done superiorly. As a consequence of these dramatic changes in the arrangement of the gross anatomy the subcortical anatomy of the white matter and the occipital horn is rendered. The occipital horn gained in width and height and has four walls as it did on the previous section. Amongst these walls the inferior one is very thin and corresponds to the lateral part of the inferior wall from the previous section. The medial part with the adjacent collateral sulcus is now the medial wall. The dorsal wall is, similar to the previous section but more prominently indented due to the dorsal forceps part. This section shows the transition of the occipital horn into the lateral ventricles.

The dorsal (1) and ventral (2) forceps part gained in volume. The ventral part projects dorsal along the inner surface of the occipital horn and is therefore only separated from the dorsal part by a thin gap. The fibres of the inner forceps layer merged with it. Additionally fibres originating from the inferior convexity are joining the forceps via the medial wall of the occipital horn. Likewise the dorsal forceps part gains volume from the now prominent layer of fibres that are ascending vertically along the lateral surface of the occipital horn (3). Both additions are cut longitudinally on this section whilst all other forceps fibres, including the small bundle at the inferior border of the occipital horn, are cut transversely.

Both sagittal layers migrated away from the medial surface of the dorsal forceps to allow the fibres destined for the splenium to pierce through. The majority of the stratum sagittale internum (4) is located lateral and to a smaller extent inferior to the occipital horn. On this section one can appreciate medial cortical fibres running dorsal and ventral to the forceps towards this layer (5). The stratum sagittale externum (6) tightened towards its base ventral to the occipital horn. Medial and dorsal to the dorsal forceps part no fibres of this layer are seen on this section. The directionality of the fibres is exactly the same as on the previous section. With the calvar avis the beak-like protrusions of both sagittal layers vanished. The white matter of the cingulate gyrus, namely the cingulum, is cut longitudinally (7) at its medial aspect where it descends behind the callosum. The cingulum is stained dark here and therefore easily differentiated. The cortical white matter layers are prominent and slightly darker in there staining. These include the strata propria of the sulcus collateralis (10), the precuneus (8), and the fissure interparietalis (9).

It should be noted that the dorsal and lateral areas of this specimens are generally darker stained compared to the rest. The reason for this irregularity might be found in the irregular hardening of the brain as well as the very strong (and therefore not necessarily even) de-staining necessitated by the intent to photograph the sections.

6. This cut is located approximately 10 mm anterior to the previous, approximately 75 mm away from the occipital pole, and anterior to the brain structures of this examination. The intent is to indicate the subsequent white matter trajectory. This section shows (i) the posterior part of the central sulcus (I) dorsally, (ii) the remnant of the Sylvian fissure (f.s.) laterally, and (iii) the callosum, fornix and the posterior part of the hippocampus medially. With regards to the sulcal anatomy, apart from the Sylvian fissure, the interparietal (i) and parallel sulcus (e.) as well as the second and third parietal sulci (s.t. II and III) are seen on the lateral convexity. On the medial surface one can appreciate the calloso-marginal sulcus (cm) dorsally and the collateral sulcus ventrally. The calcarine fissure already terminated prior to this section.

The occipital horn transitioned into the descending part of the cella lateralis of the lateral ventricle, which is only separated from the cortical surface ventrally through the fimbriae of the fornix (12) that are running into the hippocampus. The fibres of the forceps are freed from the white matter and the cortex, which were still separating it from the midline on the previous section, and are now located dorso-medially to the ventricle in the splenium. The dorsal (1) and ventral (2) part of the forceps can still be separated. The vertically ascending fibres (3) lateral to the ventricle are not part of the forceps in this section anymore but belong to the tapetum of the temporal lobe. The tapetum has a posterior protrusion and is thinned due to the descending part of the caudate nucleus, which is not visible on this section. The dorsal region of the tapetum is filled with cortical fibres that pierce the next layer (**). The fibres of the stratum sagittale internum (4) are all collected on the lateral surface of the ventricle and lateral to the tapetum. The dotted appearance in the middle of this layer (4*) is due to merging with other bundles from the lateral aspect of the stratum sagittale externum that are still darker and therefore differentiate from the fibres of the stratum sagittale internum. Under the microscope each of these bundles shows a rope-like twist around its own axis. The whole layer represents the posterior part of the base of the corona radiata and gains fibres ventrally from the temporal lobe and dorsally from the parietal lobe. The stratum sagittale externum (5) is now limited to the ventral part of the ventricle in the region of the temporal lobe and thins out as it sends fibres off to the temporal cortex. Towards the hippocampal gyrus, the stratum sends a protrusion that is long, thin, and a still indented by the collateral sulcus. The termination of this protrusion is joined by the cingulum. Lateral to the ventricle it extents barely until the Sylvian fissure as its demarcation fades away. The elongations of the corresponding layers of the stratum vertical convexitatis are the strata propria of the interparietal (9) and parallel sulcus (11) as well as the white matter of the Sylvian fissure (10), which are all darker stained. The cortex is closely approaching the corona radiata of the occipital lobe by a few millimetres at the deepest area in the Sylvian fissure. Dorsal to the splenium a transverse cut of longitudinal fibres shows the cingulum (7) reaching into the cingulate gyrus. On the previous section the cingulum was cut along its descending length. The lighter area between the layers of the interparietal sulcus and the Sylvian fissure indicate the location of the superior longitudinal bundle or arching bundle (6). Similar to the previous section, the dorsal and lateral areas of this specimen are darker stained compared to the rest.

7. This section is taken from a different series from an atrophic female brain of an elderly lady. This section clearly demonstrates the triple layering of the occipital horn, including its internal surfaces, and the area between the horn and the calcar avis (VI.). This section is also a coronal cut and is to be placed between the previous sections 4 and 5, only slightly anterior to the section 4. The corresponding photography demonstrates the medial aspects in a roughly fourfold enlargement and corresponds to the square that is indicated in the schematic diagram of the same section. The stem of the cuneus (VII.), which was still visible on the surface on section 4, withdrew here into the depth of the occipito-parietalis sulcus (o.) and the calcarine fissure (f.c.). The cross-section of the white matter is identical to section 4, only that it is possible to identify the medial forceps layer (2), the stratum sagittale internum (5), and externum (6) along the entire cross-section of the medial aspect of the occipital horn and between the horn and the calvar avis. These can all be seen with the naked eye.

The cross-section of the occipital horn is squared with the dorsal surface being formed by the dorsal forceps, the medial surface by the calcarine fissure, and the inferior surface by the collateral sulcus. The dorsal forceps (1) is rather prominent, the ventral forceps rather weak (4), whilst the medial forceps layer (2) is relatively strong and equally thick as the lateral layer (3). The majority of fibres of the stratum sagittale internum (5) are collected lateral to the ventricle, whilst the fibres of the externum are collected ventral to it (6). However, fibres of the latter are still to be found in the lingual gyrus and to a smaller degree in the stem of the cuneus. It is possible to trace a veil from both layers across the medial surface of the occipital horn with the naked eye.

8. This photography shows a coronal section through the temporal lobe of a brain that suffered a stroke. As a consequence of the stroke the occipital cortex and a part of the temporal cortex, especially the first temporal gyrus, ipsilateral to the lesion are damaged. The level of this section is comparable to section 5. The brain stem was removed prior to hardening this specimen. A ramification of the removal is that the temporal lobe anatomy was altered and the cortex shifted more medial. The area of the cut showing the corona radiata of the temporal lobe is bend medially and almost reaches the hippocampus, which caused the unusual form of the lateral horn.

The convexity of this section shows the Sylvian fissure laterally and the sulcus hippocampi (h.) medially. Within the section the following structures are evident: the parallel sulcus (e.), the second and third temporal sulci (s.t. II. and III.), and the collateral sulcus (coll.), which indents the lateral horn from the inside towards the eminentia collateralis Meckelii. The majority of the first temporal gyrus and some of the second temporal gyrus are affected by the stroke. From the second temporal gyrus a bright layer of degenerated fibres runs towards the white mater ditch.

**Plate 1:**
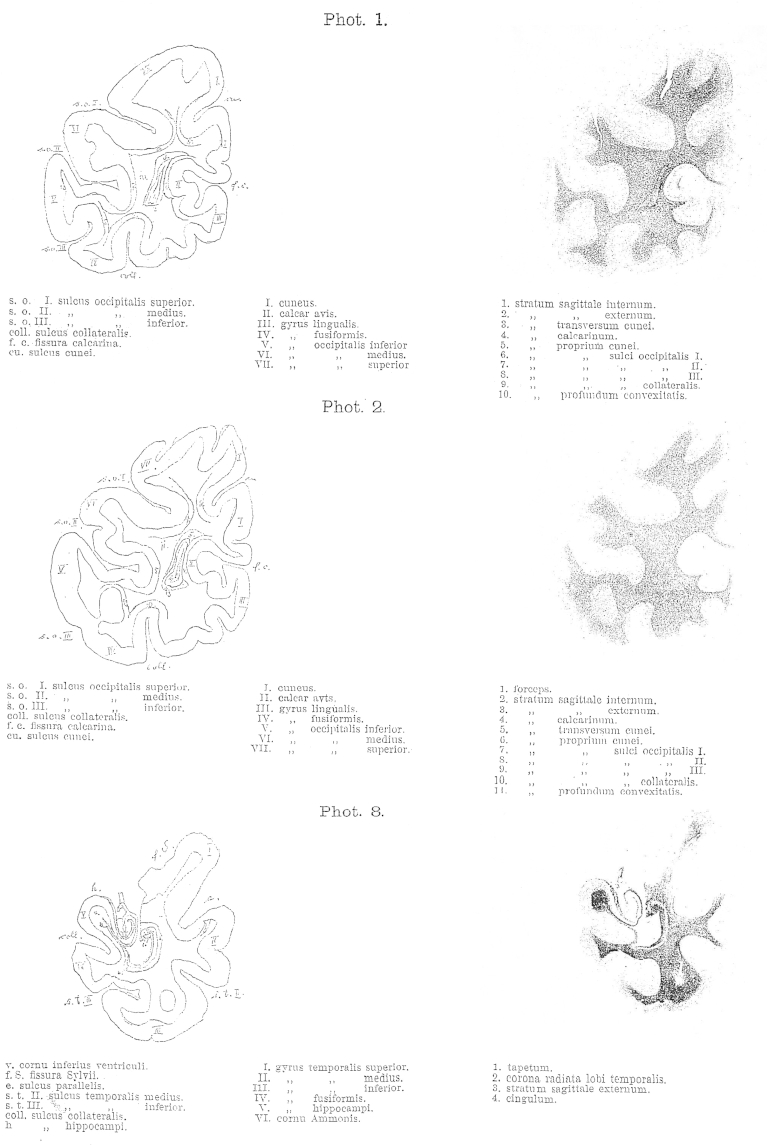


**Plate 2:**
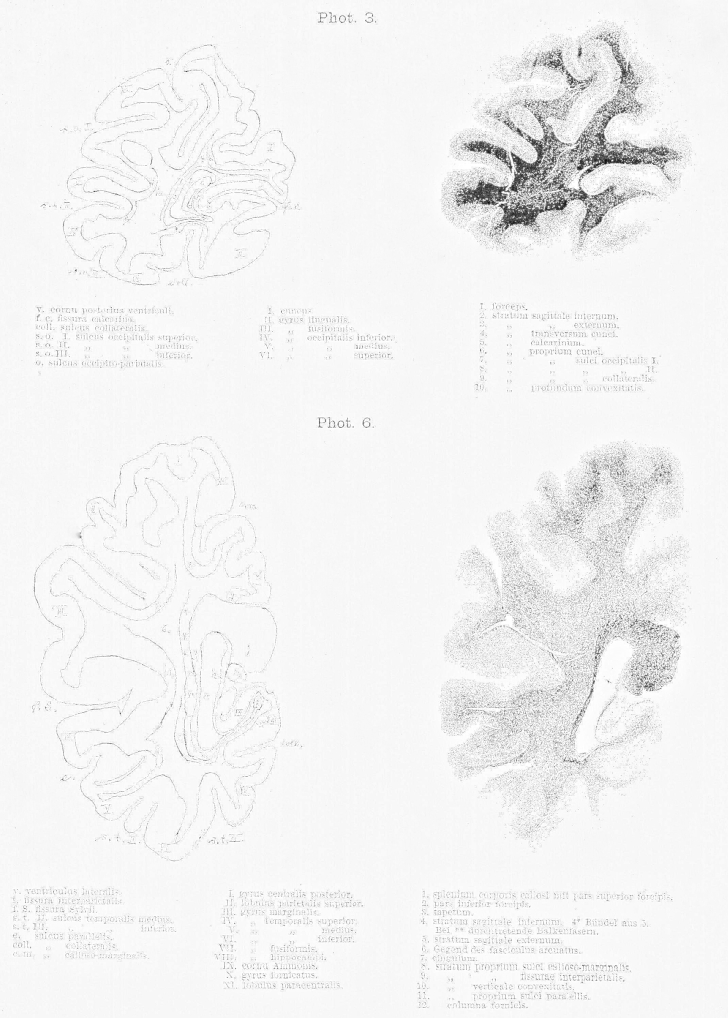


**Plate 3:**
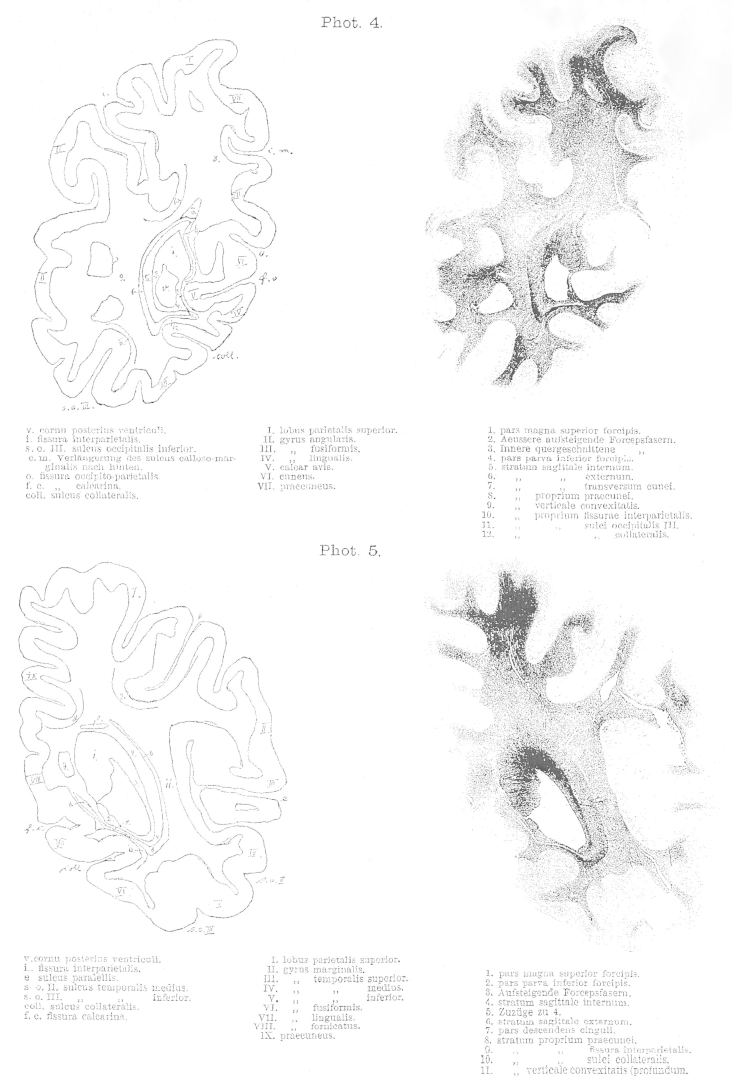


**Plate 4:**
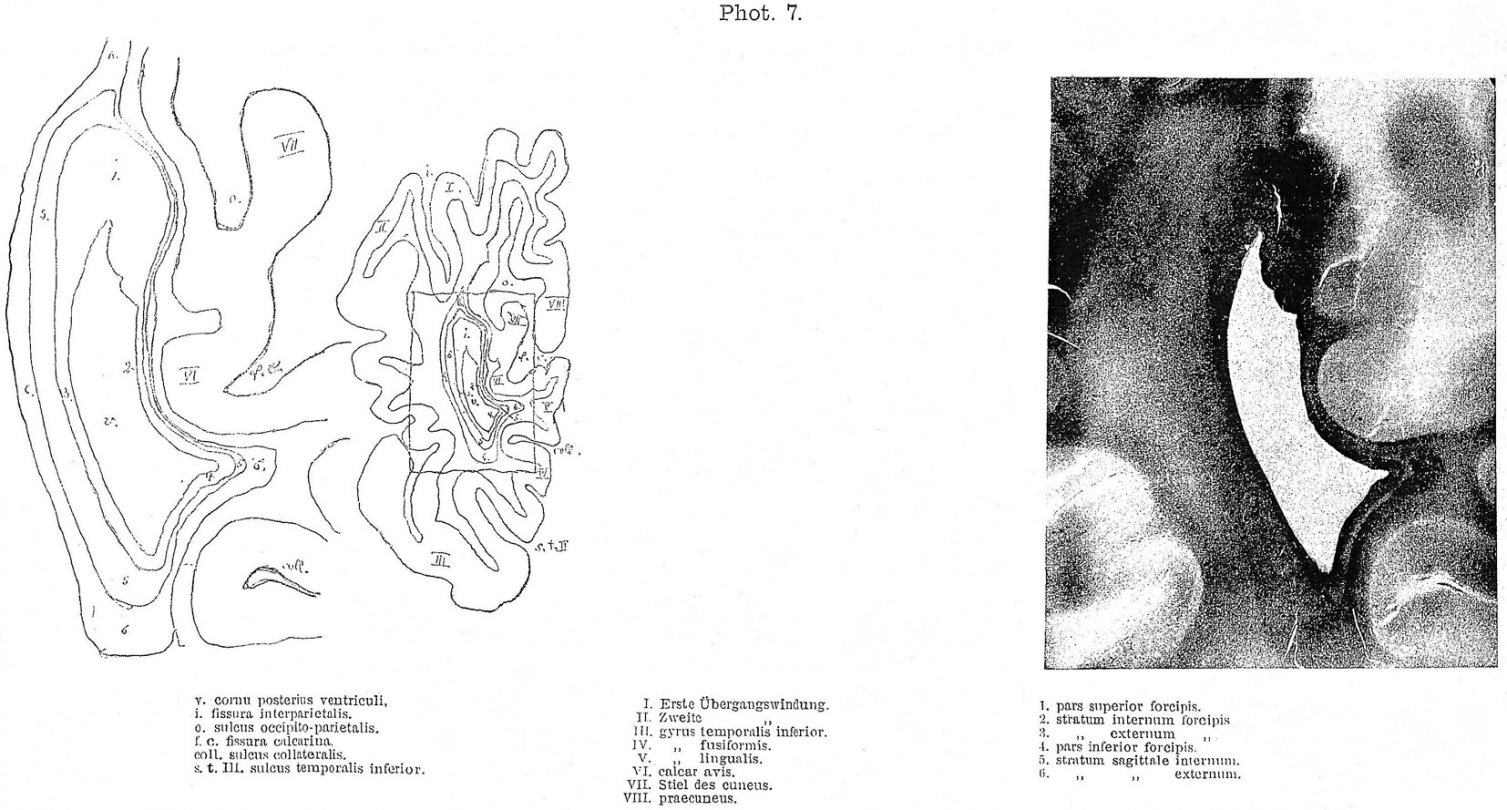


From the occipital horn a small remnant of the tapetum is present (1) lateral to it lies the well-maintained corona radiata of the temporal lobe (2) whose propagation into the elongation stratum sagittale internum is cut off. The very prominent dark transverse cut through the stratum sagittale externum, as we know it from the healthy brain, is absent. Instead, we can appreciate a bright zone with roughly the same shape, which can be followed laterally to the convexity and medially also as a small bright stripe until the hippocampal gyrus (3). Under the microscope, only insignificant remnants of white matter can be seen within this zone. The stroke of the occipital lobe therefore caused a degeneration of the entire stratum sagittale externum in the temporal lobe. A marked contrast is the cingulum in the gyrus hippocampi, which usually joins the stratum sagittale externum and is now stained deep black (Plates 1–4).

## Footnotes

2 In paralytic brains this area appears as a bright patch in degenerative cortical changes. In these cases it is focally degenerated, never in its entire length (Lissauer).

3 Note from the authors: The original text here mentions *Bogenbuendel*, which verbatim translates to *arching bundle*. When looking at Onufrowicz's original work, it becomes apparent that he equated the superior fronto-occipital fasciculus with Burdach's arcuate fasciculus: ‘This fibre tract may aptly be called ‘Fronto-occipitalis Associationsbuendel’ [associative fronto-occipital fasciculus] or could be referred to as the true fasciculus longitudinalis superior. The ingenious Burdach […] recognised, or rather guessed, this tract and called it the arcuate fasciculus or superior longitudinal fasciculus. Yet neither his [Burdach's] nor Meynert's descriptions of this tract are obvious and it is virtually impossible to prove it [fronto-occipital fasciculus] in the normal brain. We were able to appreciate this tract, located amongst callosal radiations in the normal brain only after comparison with our experiment of nature, in the case of agenesis of the corpus callosum.’ Translation in Forkel et al. (2014).
